# Supplementary material for: Early Transcriptional Responses of Human Nasal Epithelial Cells to Infection with Influenza A and SARS-CoV-2 Virus Differ and Are Influenced by Physiological Temperature
Source: Pathogens. 2023 Mar 18;12(3):480. doi: 10.3390/pathogens12030480 (PMC10051809; doi:10.3390/pathogens12030480)
Supplement: Supplementary file 1 [file pathogens-12-00480-s001.zip › supp Figure S2.pdf]

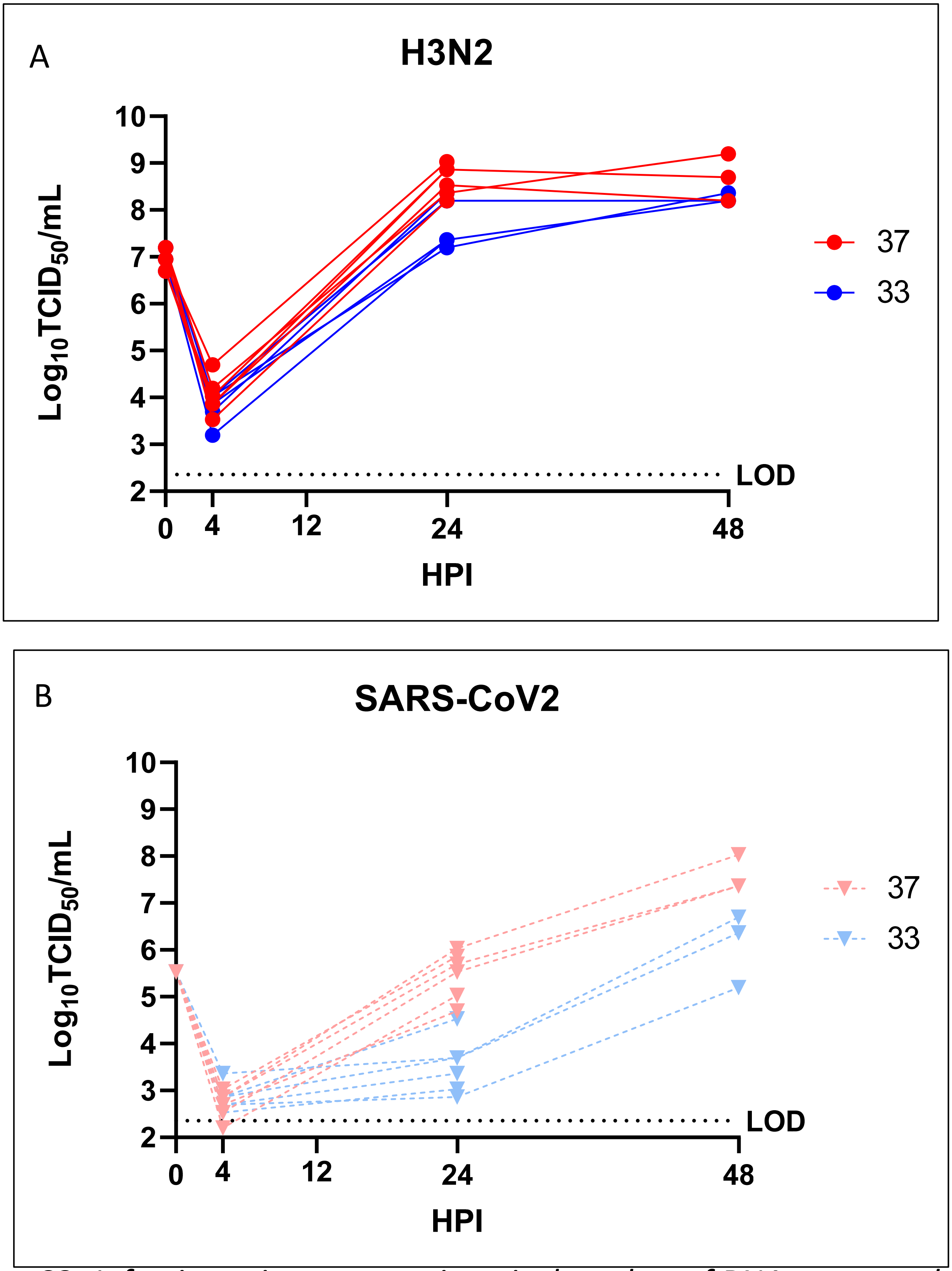

Figure S2: *Infectious virus present in apical washes of RNA-seq samples infected with SARS-CoV-2 (B) and IAV (A) viruses at 33oC or 37oC.* hNEC cultures were infected at low MOI, and followed for a multistep growth curves at 33°C and 37°C with the indicated viruses. Apical washes were taken at the timepoint indicated before sample collection. Each sample is graphed individually, and 48hr samples were also checked at 24HPI.
