# Supplementary material for: Early Transcriptional Responses of Human Nasal Epithelial Cells to Infection with Influenza A and SARS-CoV-2 Virus Differ and Are Influenced by Physiological Temperature
Source: Pathogens. 2023 Mar 18;12(3):480. doi: 10.3390/pathogens12030480 (PMC10051809; doi:10.3390/pathogens12030480)
Supplement: Supplementary file 1 [file pathogens-12-00480-s001.zip › supp Figure S3.pdf]

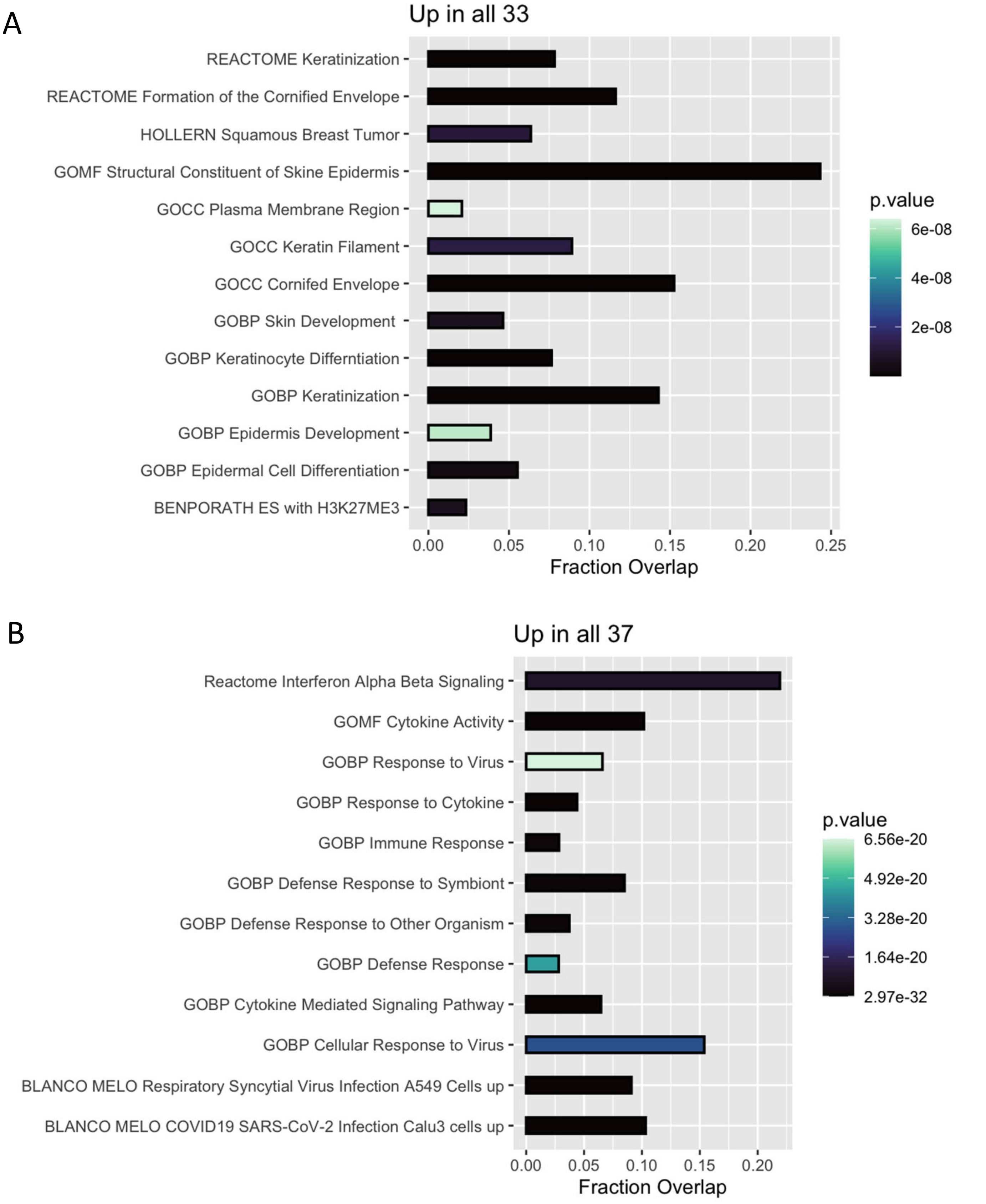

Figure S3. *Pathway analysis of differentially expressed genes between all 33°C and 37°C samples.* Differential expression analysis was run between all 33 and 37°C samples, regardless of virus treatment, and the top 250 genes were used for pathway enrichment analysis.
