# Supplementary material for: Early Transcriptional Responses of Human Nasal Epithelial Cells to Infection with Influenza A and SARS-CoV-2 Virus Differ and Are Influenced by Physiological Temperature
Source: Pathogens. 2023 Mar 18;12(3):480. doi: 10.3390/pathogens12030480 (PMC10051809; doi:10.3390/pathogens12030480)
Supplement: Supplementary file 1 [file pathogens-12-00480-s001.zip › supp Figure S6.pdf]

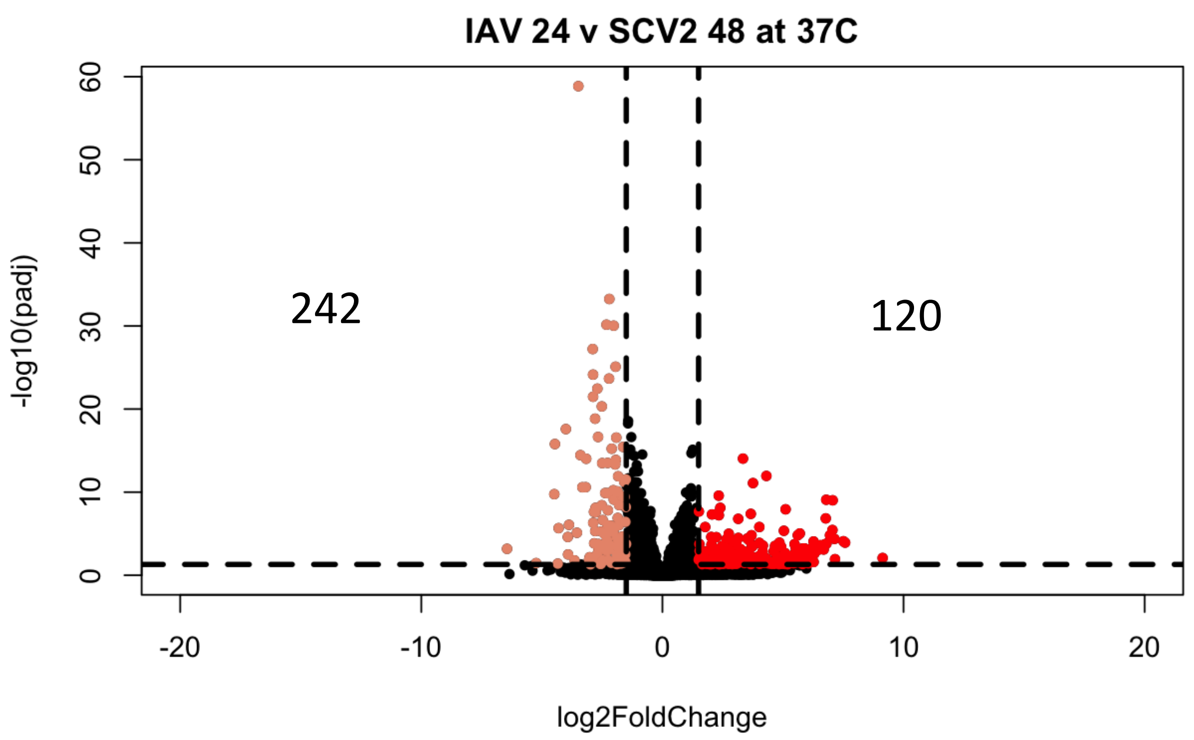

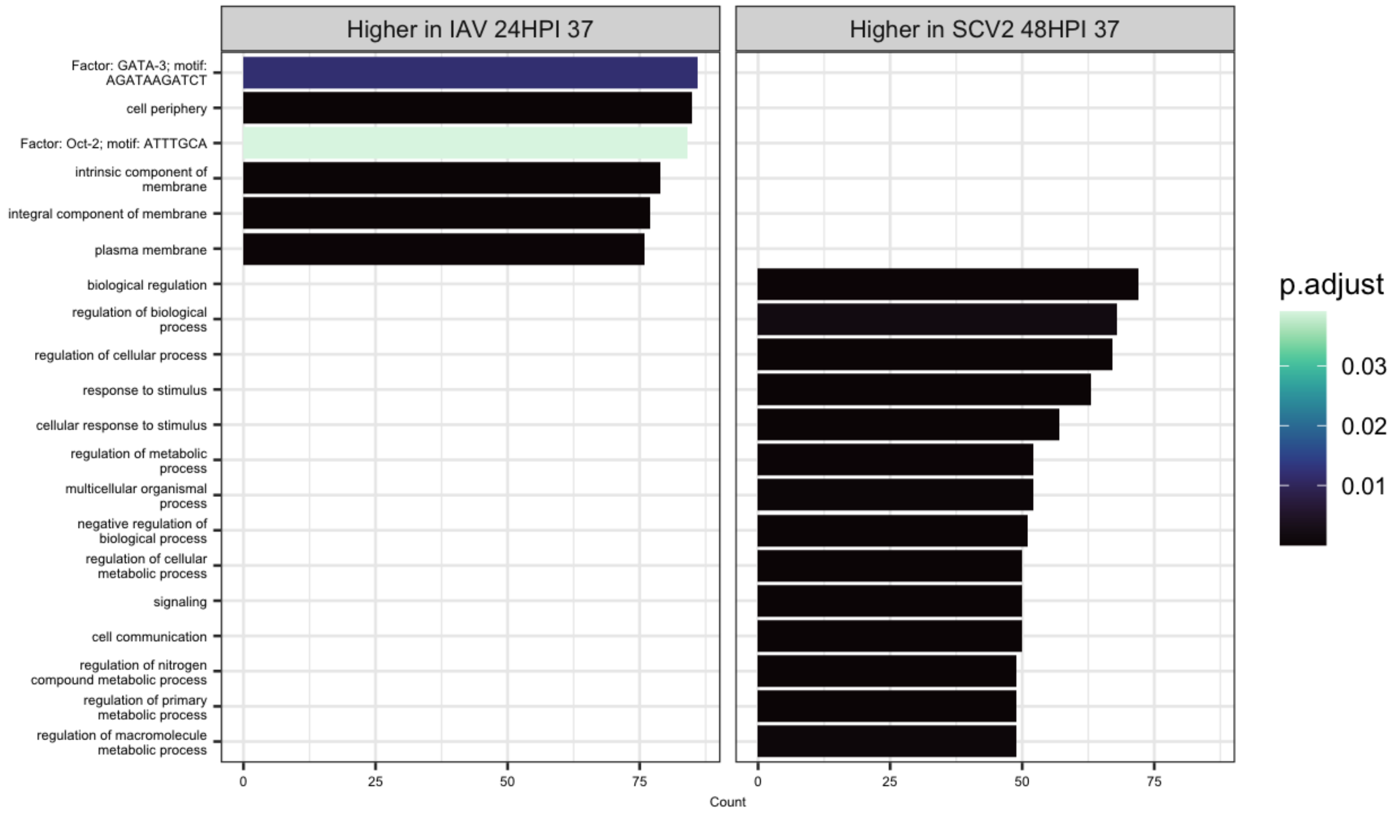

Figure S6. *Test to see if transcriptomic changes due to virus used for infection are simply delayed or are qualitatively different*. Differential expression analysis was run between IAV 24HPI and SCV2 48HPI infected samples at 37°C (A). Significantly differentially expressed genes ($p<0.05$, log2FC > 1.5) were then used in pathway enrichment analysis (B). The size of the bars indicate the number of genes in that pathway identified, and the bars are colored based on p value.
